# Arginine ameliorates motor and survival deficits in MFN2-Deficient *Drosophila* models

**DOI:** 10.1016/j.neurot.2026.e00900

**Published:** 2026-04-02

**Authors:** Masahiro Ando, Yuji Okamoto, Yujiro Higuchi, Jun-Hui Yuan, Akiko Yoshimura, Chikashi Yano, Risa Nagatomo, Takahiro Hobara, Fumikazu Kojima, Yu Hiramatsu, Satoshi Nozuma, Yusuke Sakiyama, Hiroshi Takashima

**Affiliations:** a Department of Neurology and Geriatrics, Kagoshima University Graduate School of Medical and Dental Sciences, Kagoshima, Japan; b Department of Neurology, Itoshima Medical Association Hospital, Fukuoka, Japan

**Keywords:** l-arginine, Drosophila, Rotenone, Mitochondrial dynamics, Mutation

## Abstract

Charcot–Marie–Tooth disease type 2 A (CMT2A) is an inherited axonal neuropathy linked to mutations in MFN2, a key regulator of mitochondrial dynamics. Currently, no effective drug therapies exist. l-arginine has shown promise in treating mitochondrial disorders, though its effect on MFN2-associated neuropathy remains uncertain. To investigate this, we used *Drosophila* models with the neuron-specific knockdown of Marf, the fly ortholog of MFN2, employing a temporally controlled GAL4/UAS system. Flies were administered different doses of l-arginine to examine its influence on motor ability and lifespan. To evaluate responses under mitochondrial stress, flies were also treated with rotenone, a mitochondrial complex I inhibitor. l-arginine markedly improved climbing performance under baseline conditions and extended lifespan under both baseline and stress conditions. However under rotenone-induced mitochondrial stress, high-dose l-arginine improved survival without a corresponding improvement in locomotor performance. These results support a neuroprotective role for l-arginine in MFN2-deficient *Drosophila*, possibly through effects on mitochondrial dynamics involving complex I. l-arginine may hold therapeutic promise for CMT2A, meriting further investigation in vertebrate models.

## Introduction

Charcot–Marie–Tooth (CMT) disease is among the most prevalent inherited peripheral neuropathies and is marked by progressive muscle weakness, atrophy, and sensory deficits, predominantly in the distal extremities. CMT exhibits considerable genetic heterogeneity, with over 140 causative genes identified to date [1]. These genes are associated with demyelinating (CMT1), axonal (CMT2), or intermediate forms that exhibit characteristics of both. CMT1A, which results from a duplication of the *PMP22* gene, is the most common subtype. Aside from *PMP22* duplication, the prevalence of other causative genes differs across studies [2], though *GJB1*, *MFN2*, and *MPZ* are consistently recognized as major contributors. In our prior study of Japanese patients with CMT, we observed that, excluding *PMP22* duplication, *GJB1* was the most frequently implicated gene, followed by *MFN2* [3]. Importantly, *MFN2* is the most commonly identified gene in axonal CMT (CMT2), underscoring its central role in this subtype [4,5]. *MFN2* encodes mitofusin-2, a protein located on the outer mitochondrial membrane that is critical for mitochondrial fusion and interaction with the endoplasmic reticulum [6]. Loss of *MFN2* function disrupts mitochondrial dynamics, impairs mitochondrial quality control, and leads to cellular dysfunction [7,8].

At present, no effective pharmacological treatment exists for CMT. We have concentrated on therapeutic strategies that target mitochondrial dynamics, as this area has gained interest as a promising avenue. Arginine has emerged as a potential therapeutic agent due to its reported benefits in other mitochondrial-related conditions. For example, in individuals with mitochondrial encephalomyopathy, lactic acidosis, and stroke-like episodes (MELAS), l-arginine administration has been shown to decrease the occurrence of acute attacks [9,10]. Moreover, our previous study demonstrated the effectiveness of l-arginine treatment in cases of mitochondrial myopathy with episodic hyper-creatine kinase-emia (MIMECK), indicating its possible neuroprotective role [11]. Arginine has also shown beneficial effects in diabetic neuropathy, such as increased nerve diameter and number, supporting its broader applicability in treating peripheral neuropathies [12].

In this study, we developed an MFN2 model in *Drosophila* and performed arginine administration experiments. Our results offer insights into the potential utility of arginine as a therapeutic candidate for MFN2-related CMT.

## Materials and Methods

### *Drosophila* stocks

Flies were reared in plastic vials containing standard cornmeal-yeast-agar medium at 25 °C under a 12-h light/dark cycle. Oregon R (OR) served as the wild-type control strain. The *nSyb-Gal4*, *UAS-mCD8-GFP*, and *tub-Gal80*^*ts*^ lines were obtained from the Bloomington Drosophila Stock Center (Indiana University). The *UAS-Marf-IR* (Marf RNAi) line was sourced from the Vienna Drosophila RNAi Center (transformant ID 40478).

### Induction system (GAL4/UAS/Gal80^ts^ system)

To specifically induce *Marf* RNAi in neurons during the adult stage, flies with the genotype *nSyb-Gal4/Y;* + */tub-Gal80*^*t*s^*;* and *UAS-Marf RNAi/* + were used. These flies were maintained at 18 °C and then shifted to 29 °C to activate the RNAi system before conducting behavioral and survival assays [13]. As controls, flies with the genotype *nSyb-Gal4/Y; UAS-mCD8-GFP/tub-Gal80*^*ts*^*;* + */* + were used. Control flies were also generated by crossing nSyb-Gal4, tub-Gal80^ts^ with OR, resulting in F1 progeny lacking UAS transgene expression (nSyb > +). UAS-mCD8-GFP was used as a transgenic control, whereas Oregon R (OR) was used as a wild-type background control.

### Drug administration

l-arginine at concentrations of 0.1 mg/mL (0.057 M), 1 mg/mL (0.57 M), or 10 mg/mL (5.7 M) and rotenone at concentrations of 10–50 μM were incorporated into the fly food by mixing the compounds into the medium before it solidified.

### Developmental assay (pupation and eclosion rates)

To evaluate developmental lethality, third instar larvae (n = 50 per vial) were transferred into vials containing food supplemented with either sterile water or glucose at concentrations of 1% or 10%. The number of pupae formed and adults that successfully eclosed was recorded for each condition. All experimental conditions were performed in triplicate.

### Climbing assay

Climbing ability was assessed using two different methods depending on the experiment.

For the l-arginine monotherapy experiments, climbing was evaluated as previously described [14] using a 15-cm chamber marked at 2-cm intervals. Twenty flies were tapped to the bottom, and their positions after 30 s were scored on a scale from 0 to 5 (0 = < 2 cm, 5 = ≥ 10 cm). This was repeated five times with 15-s intervals, and average scores were calculated.

For experiments involving rotenone exposure, a negative geotaxis assay was performed [15]. Twenty flies were placed in an empty vial (3-cm diameter, 20-cm height) and tapped to the bottom. The number of flies that climbed at least 5 cm within 10 s was counted. The test was repeated twice with a 15-s interval, and the mean number of successful climbers was calculated.

Two climbing protocols were used according to the experimental objectives. The graded 15-cm climbing score assay is commonly applied to assess baseline locomotor ability and is sensitive to modest motor impairment. In contrast, the 5 cm/10 s negative geotaxis assay is a simpler and widely used method for experiments involving mitochondrial toxicants such as rotenone, where acute toxicity and stress-induced motor deficits must be evaluated together. For these reasons, the negative geotaxis assay was applied in the rotenone cotreatment experiments.

### Lifespan assay

For survival analysis, 20 flies per vial were kept on standard medium at 29 °C. Flies were transferred to fresh food every 2–3 days, and the number of surviving individuals was recorded. Each experimental group consisted of 80–100 flies.

### PGC-1α manipulation

To investigate the genetic interaction between PGC-1α and MFN2, we utilized *Drosophila* lines carrying either *UAS-PGC-1α RNAi* (transformant ID #33914) or *UAS-PGC-1α* overexpression (transformant ID #20009) constructs, both obtained from the Bloomington Drosophila Stock Center. These lines were crossed with flies expressing *UAS-Marf RNAi*, and neuronal expression was driven using the same *nSyb-Gal4/tub-Gal80*^*ts*^ system described earlier. After eclosion, flies were shifted from 18 °C to 29 °C to induce gene expression. Lifespan assays were then conducted with 80–100 flies per genotype, and survival was recorded every 2–3 days.

### Statistical analyses of the Drosophila experiments

All statistical analyses were carried out using GraphPad Prism (version 10.5.0; GraphPad Software, San Diego, CA). For comparisons of developmental outcomes and climbing ability across multiple groups, the Kruskal–Wallis test followed by Dunn's multiple comparisons test was employed. Survival data were analyzed using the log-rank (Mantel–Cox) test and the Gehan–Breslow–Wilcoxon test. A p-value of <0.05 was considered statistically significant. A summary of all statistical results is provided in Table 1.

**Table 1:** Summary of statistical analyses in the MFN2 knockdown *Drosophila* models.

Experiment	Group Comparison	Variable	Statistical Test	p-value	Figure
Developmental assay	Water vs 1% glucose	Pupation %	Kruskal–Wallis + Dunn's	<0.0001	Fig. 1B
Developmental assay	Water vs 10% glucose	Pupation %	Kruskal–Wallis + Dunn's	<0.0001	Fig. 1B
Developmental assay	Control vs 0.1 mg/mL arg	Pupation %	Kruskal–Wallis + Dunn's	<0.0001	Fig. 1C
Developmental assay	Control vs 0.1 mg/mL arg	Eclosion %	Kruskal–Wallis + Dunn's	0.0108	Fig. 1C
Developmental assay	Control vs 10 mg/mL arg	Pupation %	Kruskal–Wallis + Dunn's	0.0497	Fig. 1E
Developmental assay	Control vs 10 mg/mL arg	Eclosion %	Kruskal–Wallis + Dunn's	0.0289	Fig. 1E
Climbing assay (Day11, arg)	Control vs 0.1 mg/mL arg	Climbing ability	Kruskal–Wallis + Dunn's	<0.0001	Fig. 2C
Climbing assay (Day11, arg)	Control vs 10 mg/mL arg	Climbing ability	Kruskal–Wallis + Dunn's	0.0239	Fig. 2C
Climbing assay (Day 17, arg)	Control vs 1 mg/mL arg	Climbing ability	Kruskal–Wallis + Dunn's	<0.001	Fig. 2D
Lifespan assay (arg 10 mg/mL)	Control vs arg	Survival	Gehan–Breslow–Wilcoxon	0.0256	Fig. 2E
Climbing assay (Day11, rot)	All conditions∗	Climbing %	Kruskal–Wallis	<0.0001	Fig. 3A
Climbing assay (Day11, rot)	Control vs MFN2 KD/25 μM rotenone	Climbing %	Kruskal–Wallis + Dunn's	0.178	Fig. 3A
Climbing assay (Day11, rot)	Control vs MFN2 KD/50 μM rotenone	Climbing %	Kruskal–Wallis + Dunn's	0.0377	Fig. 3A
Lifespan assay (rot/Arg)	Control vs 25 μM rotenone	Survival	Log-rank	<0.0001	Fig. 3B
Lifespan assay (rot/Arg)	25 μM rotenone vs 10 mg/mL arg	Survival	Log-rank	<0.0001	Fig. 3B
Climbing assay (rot/Arg)	MFN2 KD + rot vs MFN2 KD + rot + arg	Climbing %	Two-way ANOVA	n.s.	Fig. 3C
Lifespan assay (MFN2/PGC-1α)	Control vs PGC-1α KD	Survival	Log-rank/GBW	<0.0001	Fig. S1B
Lifespan assay (MFN2/PGC-1α)	PGC-1α KD vs MFN2 KD + PGC-1α KD	Survival	Log-rank/GBW	<0.0001	Fig. S1B
Lifespan assay (MFN2/PGC-1α)	Control vs PGC-1α OE	Survival	Log-rank/GBW	<0.0001	Fig. S1D
Lifespan assay (MFN2/PGC-1α)	PGC-1α OE vs MFN2 KD + PGC-1α OE	Survival	Log-rank/GBW	<0.0001	Fig. S1D

Abbreviations: Arg, l-arginine; Rot, rotenone; OE, overexpression; KD, knockdown; GBW, Gehan–Breslow–Wilcoxon; n.s., not significant.

∗Kruskal–Wallis test was applied to all five groups: nSyb > GFP, nSyb > GFP +50 μM rotenone, nSyb > Mfn +10 μM rotenone, nSyb > Mfn +25 μM rotenone, and nSyb > Mfn +50 μM rotenone.

∗∗ Two-way ANOVA was used to evaluate the effects of arginine treatment and time.

## Results

### Lethality assessment in MFN2 knockdown flies using w; +; elav-GAL4/UAS-Marf-IR

**Fig. 1:**
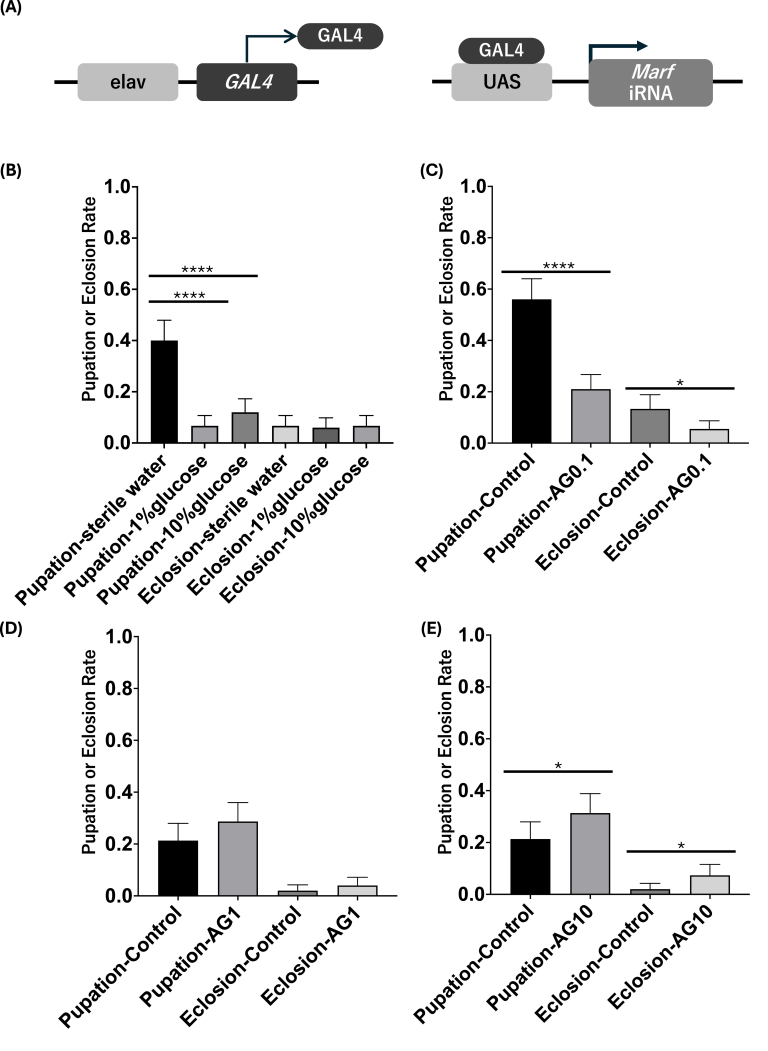
Arginine treatment enhances pupation and eclosion rates in MFN2 knockdown flies **(a)** Diagram of the experimental design. Neuron-specific Marf knockdown (the *Drosophila* ortholog of MFN2) was achieved using elav-GAL4 > UAS-Marf-IR. Flies were reared on diets supplemented with either glucose or varying concentrations of arginine **(b)** Pupation and eclosion rates under different glucose concentrations. Glucose feeding significantly reduced pupation compared to water-fed controls. Eclosion rates remained low in all groups **(c–e)** Effects of arginine at 0.1 mg/mL, 1 mg/mL, and 10 mg/mL on pupation and eclosion. High-dose arginine (10 mg/mL) significantly improved both outcomes relative to controls Data are presented as mean ± 95% confidence interval. ∗p < 0.05, ∗∗∗∗p < 0.0001, based on Kruskal–Wallis test with Dunn's post hoc comparisons. AG, l-arginine.

To assess the impact of MFN2 knockdown on developmental viability, we examined pupation and eclosion rates under various dietary conditions using *w;* + *; elav-GAL4/UAS-Marf-IR* flies (Fig. 1a and b). In this system, the neuron-specific *elav* promoter drives *GAL4* expression, which in turn activates UAS-mediated RNAi targeting *Marf*, thereby selectively reducing MFN2 function in neurons. In the control group fed with sterile water, the median pupation rate was 40%. In contrast, the groups fed with glucose showed significantly lower pupation rates: 6.7% with 1% glucose and 12% with 10% glucose. Statistical analysis using the Kruskal–Wallis test followed by Dunn's multiple comparisons indicated significant differences between the glucose-fed and control groups (p < 0.0001), while no significant difference was observed between the two glucose concentrations. Conversely, eclosion rates were consistently low across all feeding conditions, and no statistically significant differences were identified (Kruskal–Wallis, p = 0.9639).

### Improvement of pupation and eclosion rates by arginine administration

To assess the therapeutic potential of arginine, we investigated the effects of various concentrations (0.1 mg/mL, 1 mg/mL, and 10 mg/mL) on pupation and eclosion rates in MFN2 knockdown flies. At 0.1 mg/mL, both pupation and eclosion rates were significantly lower than those of the control group (p < 0.0001 and p = 0.0108, respectively) (Fig. 1c). At 1 mg/mL, there was a slight improvement in both rates, though the differences were not statistically significant (p = 0.1434) (Fig. 1d). Importantly, administration of 10 mg/mL arginine led to a significant increase in both pupation and eclosion rates compared to the control group (p = 0.0497 and p = 0.0289, respectively), suggesting that a high dose of arginine may partially alleviate the developmental lethality observed in MFN2 knockdown flies (Fig. 1e). All data are presented as mean ± 95% confidence interval.

### Effect of arginine on climbing ability and survival in the MFN2 model flies

**Fig. 2:**
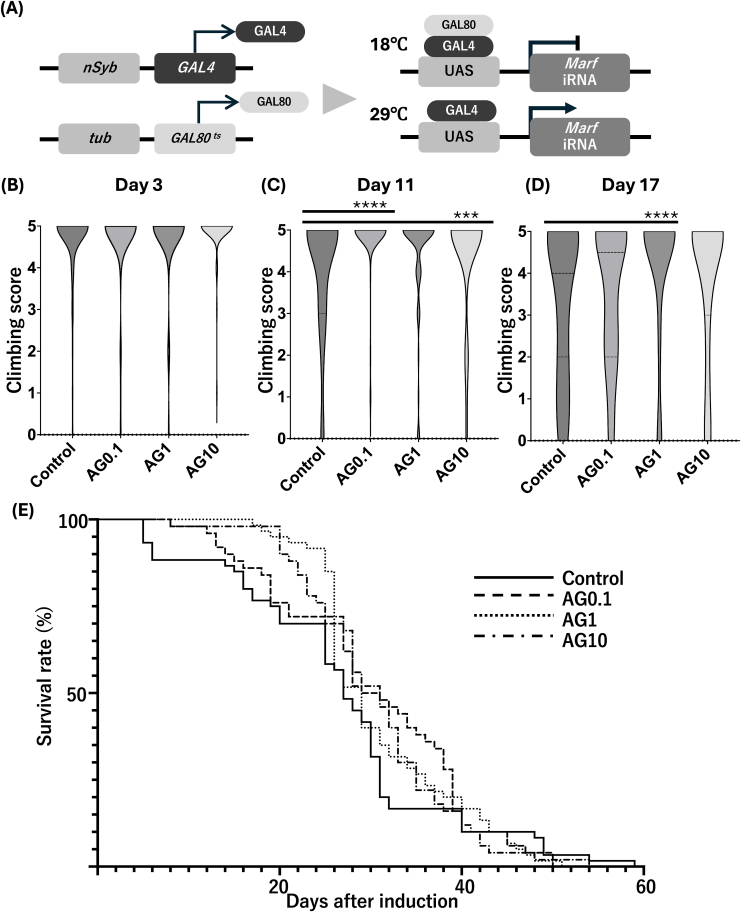
Arginine improves motor function and survival in adult MFN2 knockdown flies **(a)** Schematic of temporal control using the Gal4/UAS/gal80^ts^ system. Marf RNAi was activated in adult neurons by shifting from 18 °C to 29 °C after eclosion **(b–d)** Climbing performance was assessed on days 3, 11, and 17 following RNAi induction. Climbing scores were recorded for flies treated with control or AG at 0.1, 1, or 10 mg/mL. Data are shown as mean ± 95% confidence interval (e) Survival curves of MFN2 knockdown flies given control or varying doses of AG. A significant survival benefit was observed in the 10 mg/mL group compared to control (Gehan–Breslow–Wilcoxon test, p = 0.0256) ∗∗∗p < 0.001, ∗∗∗∗p < 0.0001. AG, l-arginine.

To evaluate functional recovery in adult flies with neuron-specific MFN2 knockdown, we utilized the Gal4/UAS/gal80^ts^ system to control RNAi induction through temperature regulation (Fig. 2a). At 18 °C, GAL4 activity was suppressed by GAL80^ts^, while shifting the temperature to 29 °C after eclosion permitted neuronal expression of MARF RNAi.

Climbing ability was assessed at three time points: day 3, day 11, and day 17. On day 3, no significant differences were observed among the groups (p = 0.5065) (Fig. 2b). By day 11, the control group exhibited a marked decline in climbing ability, whereas flies treated with 0.1 mg/mL and 10 mg/mL arginine showed significant improvement compared to control (p < 0.0001 and p = 0.0239, respectively). In contrast, 1 mg/mL arginine had no significant effect at this stage (Fig. 2c). On day 17, climbing ability in the control group declined further. Of the arginine-treated groups, 1 mg/mL arginine produced the most notable improvement relative to the control (p < 0.001), while 10 mg/mL arginine showed a trend toward improvement without reaching statistical significance. The 0.1 mg/mL group showed no significant difference from the control (Fig. 2d).

To assess the long-term impact of arginine treatment, survival analysis was performed. Although the log-rank test did not indicate significant differences, the Gehan–Breslow–Wilcoxon test identified a modest but statistically significant increase in survival in the 10 mg/mL group compared to the control (p = 0.0256). Median survival increased from 27 days in the control group to 31 days with 10 mg/mL arginine (Fig. 2e).

### Rescue of climbing ability and survival by arginine in rotenone-treated MFN2 model flies

**Fig. 3:**
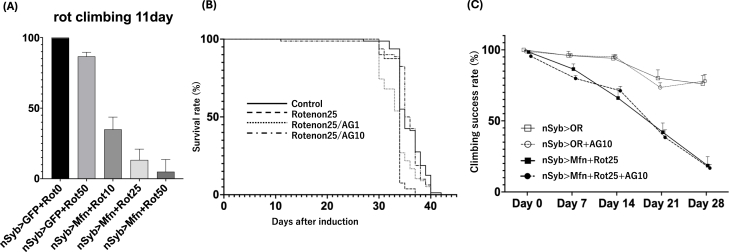
Rescue of motor and survival deficits in MFN2 knockdown flies under mitochondrial stress via rotenone **(a)** Day 11 climbing performance in MFN2 knockdown flies and controls using Gal4/UAS/gal80^ts^ system (nSyb > Marf RNAi (Mfn), nSyb > GFP) exposed to 0, 10, 25, or 50 μM rotenone. In the 50 μM group, deceased flies were included in the total to calculate successful climbing percentages. MFN2 knockdown flies exhibited a dose-dependent motor deficit, whereas controls maintained performance even at 50 μM **(b)** Survival curves of MFN2 knockdown flies with 25 μM rotenone and cotreatment with either 1 or 10 mg/mL l-arginine (AG). Rotenone significantly reduced survival, which was markedly rescued by 10 mg/mL AG (log-rank test, p < 0.0001) (**c)** Locomotor performance of flies generated using Gal4/UAS/gal80^ts^ system, including wild-type background controls (nSyb>+; Oregon R (OR)) and MFN2 knockdown flies (nSyb > Marf RNAi (Mfn)) treated with rotenone (25 μM) with or without AG (10 mg/mL). AG treatment did not significantly improve climbing performance in MFN2 knockdown flies exposed to rotenone. UAS-GFP was used as a transgenic control, whereas Oregon R (OR) was used as a wild-type background control.

To investigate the role of mitochondrial complex I dysfunction in MFN2 model flies, we administered rotenone, a known complex I inhibitor [16]. A climbing assay was conducted on day 11 using MFN2 knockdown flies (*nSyb > Marf RNAi*) and control flies (*nSyb > GFP*), which were treated with 0, 10, 25, or 50 μM rotenone. In the 50 μM group, some flies died prior to testing. To maintain consistency across groups, the percentage of successful climbers was calculated based on the initial number of flies, including those that had died. The results revealed a dose-dependent decrease in climbing ability among the MFN2 knockdown flies, while control flies maintained normal performance even at 50 μM (Fig. 3a). Given the observed toxicity at 50 μM, which led to premature death in a notable proportion of flies, 25 μM was selected as the optimal dose for subsequent experiments, including the lifespan assay to evaluate the therapeutic effects of l-arginine.

Treatment with 25 μM rotenone significantly reduced survival in the MFN2 knockdown flies compared with untreated controls (p < 0.0001, log-rank test). Coadministration of 1 mg/mL arginine resulted in only a slight, insignificant improvement (p = 0.7892), whereas 10 mg/mL arginine significantly improved survival to a level comparable with untreated controls (p < 0.0001, log-rank test) (Fig. 3b).

To evaluate whether l-arginine mitigates locomotor impairment under mitochondrial stress, climbing performance was assessed in MFN2 knockdown flies exposed to rotenone with or without l-arginine treatment. Rotenone exposure resulted in a marked decline in locomotor performance. l-arginine treatment did not significantly improve climbing performance compared with rotenone treatment alone (Fig. 3C), despite the observed improvement in survival.

### Genetic interaction between PGC-1α and MFN2 in MFN2 model flies

PGC-1α plays a central role in regulating mitochondrial biogenesis and energy metabolism [17]. To explore the genetic interaction between MFN2 and PGC-1α, we assessed survival in flies with either PGC-1α knockdown or overexpression, both alone and in combination with MFN2 knockdown. PGC-1α knockdown significantly reduced survival, which was further decreased when combined with MFN2 knockdown, suggesting an additive detrimental effect (Supplementary Fig. 1A and B). Although PGC-1α overexpression alone reduced survival, its coexpression with MFN2 knockdown significantly improved survival compared with PGC-1α overexpression alone, suggesting a partial mitigating effect (Supplementary Fig. 1C and D).

## Discussion

In this study, we demonstrated that arginine administration provides therapeutic benefits in *Drosophila* models with MFN2 deficiency. Our results indicate that high-dose arginine improves developmental parameters such as pupation and eclosion rates and alleviates motor impairment under baseline conditions and improves survival in adult flies with neuron-specific MFN2 knockdown. These findings support the potential of arginine as a therapeutic option for MFN2-related neuropathies, possibly through effects on mitochondrial function.

MFN2 is essential for mitochondrial fusion and maintaining mitochondrial quality control. Unlike mammals, *Drosophila* does not possess an MFN1 homolog, rendering MFN2 loss particularly detrimental [18]. Prior research using CMT2 model flies has proposed both gain-of-function and loss-of-function mechanisms, reflecting the complexity of MFN2-associated disease pathology [19]. In our study, we employed a severe knockdown model to specifically examine the effects of MFN2 deficiency. Loss of MFN2 disrupts mitochondrial dynamics, leading to impaired energy production and elevated oxidative stress. Administration of high-dose arginine partially rescued developmental viability, as shown by the increased pupation and eclosion rates. This outcome may be related to arginine's known role in promoting nitric oxide (NO) synthesis and supporting mitochondrial activity—both critical for maintaining cellular energy metabolism [20].

Because MFN2 knockdown results in severe lethality, direct assessment of adult motor function under standard conditions was not possible. To address this issue, we utilized the Gal4/UAS/gal80^ts^ system, which enables temporal control of RNAi induction following eclosion. By shifting the temperature after adult emergence, MFN2 knockdown was limited to the adult stage. Climbing assays showed that motor function declined over time in control flies, whereas arginine treatment produced improvements that were both dose- and time-dependent. On day 11, climbing ability significantly improved with 0.1 mg/mL and 10 mg/mL arginine; by day 17, 1 mg/mL arginine showed the most pronounced effect. Our findings reveal a complex, context- and dose-dependent effect of l-arginine in MFN2-deficient flies. Under basal mitochondrial stress, low (0.1 mg/mL) and high (10 mg/mL) doses improved the phenotype at day 11, whereas the intermediate dose (1 mg/mL) was initially ineffective. By day 17, the intermediate dose produced the most pronounced rescue, while the low and high doses no longer reached significance. These findings are consistent with previously reported neuroprotective properties of arginine in other models of neuropathy, such as diabetic neuropathy [12]. In contrast, under acute complex I stress induced by rotenone, the highest dose conferred the strongest survival benefit. These observations suggest that l-arginine's therapeutic efficacy depends on both treatment timing and the severity of mitochondrial stress. The non-monotonic, time-dependent response may reflect a combination of hormetic effects, saturation or feedback within protective pathways, and differential metabolic processing or substrate availability, which together influence the engagement of downstream neuroprotective mechanisms. Collectively, these results highlight the importance of carefully titrated dosing in strategies aimed at mitigating mitochondrial dysfunction.

In survival analysis, 10 mg/mL arginine significantly enhanced early-phase survival, as indicated by the Gehan–Breslow–Wilcoxon test. Although the log-rank test did not yield a statistically significant result, the early-phase benefit implies that arginine may help reduce the initial susceptibility associated with MFN2 deficiency.

To further examine the protective role of arginine under heightened mitochondrial stress conditions, we treated MFN2 knockdown flies with rotenone, a complex I inhibitor. Rotenone administration significantly decreased survival, intensifying the mitochondrial dysfunction associated with MFN2 deficiency. While high-dose arginine (10 mg/mL) significantly improved survival, the low-dose (1 mg/mL) did not yield a comparable effect. These results suggest that arginine's therapeutic benefit may be closely associated with its capacity to support complex I activity, possibly through NO-related mechanisms or by enhancing mitochondrial energy metabolism. Although locomotor performance was assessed under rotenone exposure, arginine treatment did not significantly improve motor function. This dissociation between survival benefit and functional recovery suggests that arginine may enhance neuronal resilience under mitochondrial stress without fully restoring neuromuscular function. These findings further indicate that metabolic support alone may be insufficient to reverse established motor deficits. It is also possible that the combined mitochondrial stress imposed by MFN2 deficiency and rotenone exposure exceeded the capacity of metabolic support to restore motor function.

We also evaluated the role of PGC-1α, a central regulator of mitochondrial biogenesis [17]. Knockdown of PGC-1α alone significantly shortened survival, and the combined knockdown of MFN2 and PGC-1α resulted in an even greater reduction, suggesting an additive negative impact on neuronal survival. In contrast, although PGC-1α overexpression alone reduced survival, its coexpression with MFN2 knockdown significantly extended lifespan compared to PGC-1α overexpression alone, suggesting a genetic interaction between these pathways. Although the present study does not establish a role for PGC-1α in l-arginine–mediated protection, the findings indicate that mitochondrial regulatory pathways may modulate the severity of MFN2-associated phenotypes and warrant further investigation.

A limitation of this study is the use of an MFN2 knockdown *Drosophila* model, which likely presents a more severe phenotype than that typically seen in CMT2A patients. Although both gain-of-function and loss-of-function mechanisms have been implicated in CMT2A, our model does not include MFN2 point mutations, which may more closely mimic clinical manifestations. Future research should employ models based on point mutations to more accurately capture the genetic and mechanistic heterogeneity of MFN2-associated neuropathies.

Moreover, since all experiments were performed in *Drosophila*, the translational applicability of our findings remains to be determined. To evaluate the clinical potential of arginine treatment, it will be necessary to validate these results in other model organisms, including vertebrate systems, and eventually in human studies.

This study provides evidence supporting arginine as a potential therapeutic strategy for MFN2-related CMT disease, possibly through its effects on mitochondrial dynamics and complex I-related stress. Further investigations are needed to elucidate the molecular mechanisms underlying its neuroprotective effects and to assess its relevance in clinical applications.

## Author contributions

MA, YO, and HT conceived and designed the study. MA, YO, YH, AY, CY, RN, TH, FK, YH, SN, and YS contributed to the acquisition and analysis of Drosophila experimental data. MA, AY, JY, JM, and ST contributed to the genetic analysis. JY performed English language editing. MA drafted the manuscript. All authors reviewed and approved the final version of the manuscript.

## Data availability

Datasets are not readily available due to ethical and privacy restrictions. Requests should be directed to the corresponding author.

## Funding

This study was partly supported by a Grant-in-Aid from the Research Committee of 10.13039/501100000346Ataxia, 10.13039/100018696Health Labor Sciences Research Grant, the 10.13039/100009647Ministry of Health, Labor and 10.13039/100018696Health, Welfare and Labor, Japan (2016100002B). This research was supported by the 10.13039/100009619Japan Agency for Medical Research and Development (Grant Numbers 201442014A and 201442071A). This research was also supported by 10.13039/501100001691JSPS KAKENHI (Grant Numbers JP18H02742, JP20K16604, JP21K15702, JP21H02842, JP22K15713, JP22K07495, JP22K07519, JP23K06931, and JP23K06966, JP24K18708).

## Declaration of competing interest

The authors declare the following financial interests/personal relationships which may be considered as potential competing interests:

Higuchi Yujiro reports financial support was provided by JSPS KAKENHI. Masahiro Ando reports financial support was provided by JSPS KAKENHI. JunHui Yuan reports financial support was provided by JSPS KAKENHI. Yuji Okamoto reports financial support was provided by JSPS KAKENHI. Akiko Yoshimura reports financial support was provided by JSPS KAKENHI. Yu Hiramatsu reports financial support was provided by JSPS KAKENHI. Hiroshi Takashima reports financial support was provided by JSPS KAKENHI, AMED. If there are other authors, they declare that they have no known competing financial interests or personal relationships that could have appeared to influence the work reported in this paper.
