# Supplementary figures and images for: Arginine ameliorates motor and survival deficits in MFN2-Deficient Drosophila models
Source: Neurotherapeutics. 2026 Apr 2;23(3):e00900. doi: 10.1016/j.neurot.2026.e00900 (PMC13084675; doi:10.1016/j.neurot.2026.e00900)

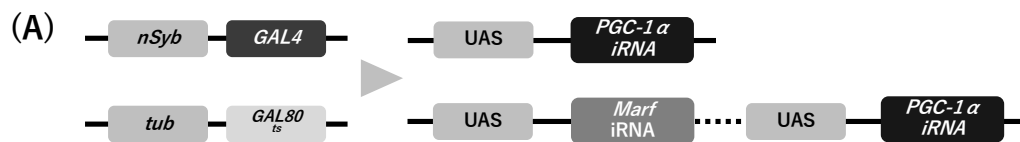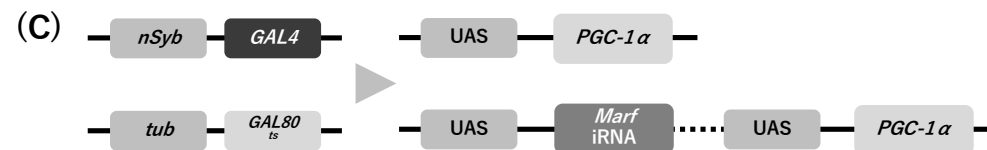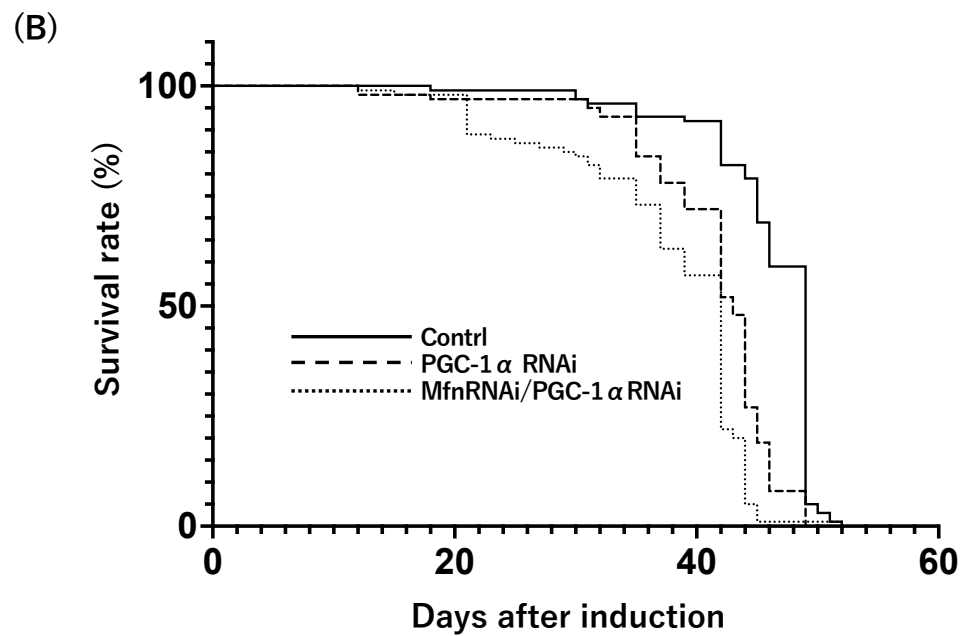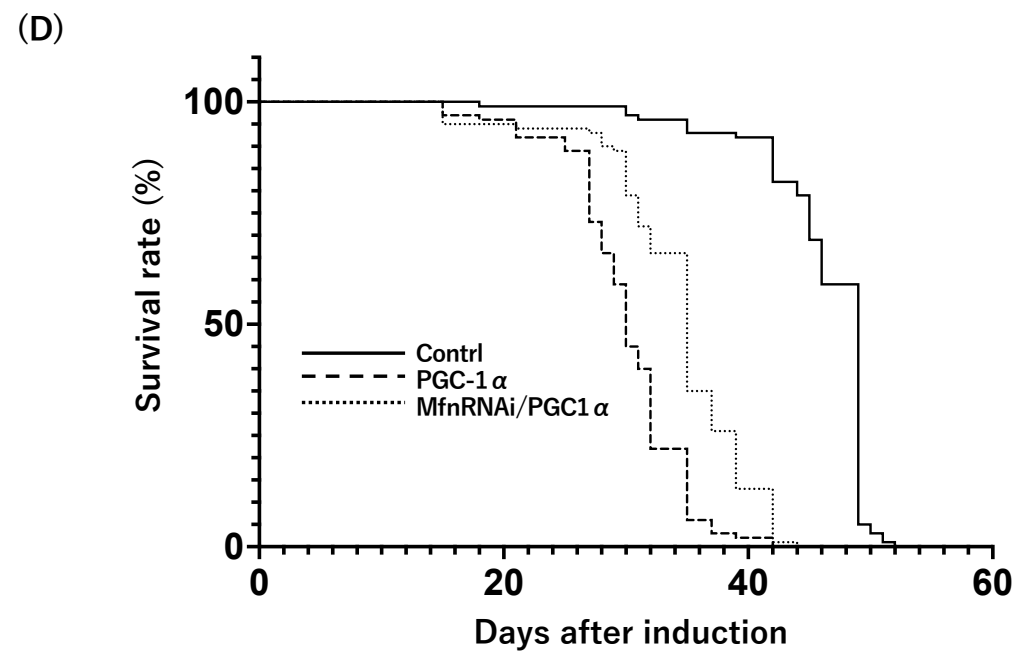

Supplement: Multimedia component 1 [file mmc1.pdf]
